# Investigating mycobacterial topoisomerase I mechanism from the analysis of metal and DNA substrate interactions at the active site

**DOI:** 10.1093/nar/gky492

**Published:** 2018-06-14

**Authors:** Nan Cao, Kemin Tan, Thirunavukkarasu Annamalai, Andrzej Joachimiak, Yuk-Ching Tse-Dinh

**Affiliations:** 1 Department of Chemistry and Biochemistry, Florida International University, 11200 SW 8 St, Miami, FL 33199, USA; 2 Biomolecular Sciences Institute, Florida International University, 11200 SW 8 St, Miami, FL 33199, USA; 3 Structural Biology Center, Biosciences, Argonne National Laboratory, 9700 S. Cass Avenue, Argonne, IL 60439, USA

## Abstract

We have obtained new crystal structures of *Mycobacterium tuberculosis* topoisomerase I, including structures with ssDNA substrate bound to the active site, with and without Mg^2+^ ion present. Significant enzyme conformational changes upon DNA binding place the catalytic tyrosine in a pre-transition state position for cleavage of a specific phosphodiester linkage. Meanwhile, the enzyme/DNA complex with bound Mg^2+^ ion may represent the post-transition state for religation in the enzyme's multiple-step DNA relaxation catalytic cycle. The first observation of Mg^2+^ ion coordinated with the TOPRIM residues and DNA phosphate in a type IA topoisomerase active site allows assignment of likely catalytic role for the metal and draws a comparison to the proposed mechanism for type IIA topoisomerases. The critical function of a strictly conserved glutamic acid in the DNA cleavage step was assessed through site-directed mutagenesis. The functions assigned to the observed Mg^2+^ ion can account for the metal requirement for DNA rejoining but not DNA cleavage by type IA topoisomerases. This work provides new structural insights into a more stringent requirement for DNA rejoining versus cleavage in the catalytic cycle of this essential enzyme, and further establishes the potential for selective interference of DNA rejoining by this validated TB drug target.

## INTRODUCTION

*Mycobacterium tuberculosis* is the chief causative agent of tuberculosis (TB), the leading cause of morbidity and mortality globally with an estimated 10.4 million cases and 1.8 million deaths annually as reported by WHO in 2017. *M. tuberculosis* topoisomerase I (MtbTOP1) is a validated target for much needed new antibiotics that can be used against the MDR-TB (multidrug-resistant tuberculosis) cases that are difficult to treat (1–3). Topoisomerases control the topological state of the genome, thus affecting vital cellular processes including replication, transcription, recombination and repair (4–6). Type I topoisomerases catalyze DNA topological transformations by breaking and rejoining a single strand of DNA at a time, and are divided into type IA, type IB and type IC subfamilies based on distinctively different protein sequences and catalytic mechanisms (4,5,7,8). MtbTOP1 and topoisomerase I present in all bacterial pathogens are type IA topoisomerases that are needed for resolving topological barriers that require the passage of DNA through the break created on single-stranded DNA (ssDNA) (6,9). This can be exploited for bactericidal action with topoisomerase poison inhibitors (10–12) that can stabilize the break by inhibiting DNA rejoining. The bacterial type IIA topoisomerases have been targeted extensively for discovery of antibiotics that include the widely used fluoroquinolones (13,14). The efforts to discover bacterial topoisomerase I inhibitors would be aided greatly by the knowledge of how DNA religation following formation of the covalent intermediate can be inhibited.

Type IA topoisomerases share certain functional and structural elements of similarities with type IIA topoisomerases (15), even though these two classes of topoisomerases appear to have evolved independently as essential topoisomerase activities in living organisms (9). Both type IA and type IIA topoisomerases form a 5′-phosphoryl tyrosine linkage with cleaved DNA in a covalent intermediate (16), and divalent metal ions are absolutely required for the change in topology catalyzed by these topoisomerases (7). However, there is a significant difference in the biochemical mechanism of these two classes of topoisomerases. Type IA topoisomerases can cleave DNA in the absence of divalent metal ions to form a covalent intermediate (17), while DNA cleavage by type IIA topoisomerases requires the addition of divalent metal ions (18). The requirement of metal ions for DNA religation, but not for DNA cleavage by bacterial topoisomerase I may present an opportunity for selective inhibition of DNA religation by small molecule inhibitors. None of the previously determined structures of type IA topoisomerases has divalent metal ions and DNA both present at the active site. The role of metal ions in the catalytic mechanism of bacterial topoisomerase I needs to be clarified further by structural information.

Here, we present new structures of MtbTopI determined by X-ray crystallography. For the first time, divalent metal ion can be observed to coordinate DNA, water molecules, and the acidic functional groups in the active site of a type IA topoisomerase. We observed metal ion interactions with the aspartates in the DxD motif (D111 and D113), proposed previously based on genetic and biochemical data (19–21). The invariant glutamic acid (E24) in the TOPRIM domain is in a position for key roles in the catalysis of DNA cleavage-religation. The requirement of the carboxyl group on E24 for acid-base catalysis of DNA cleavage in the absence of divalent metal ions is further clarified by study of the E24A and E24Q mutants. Based on the new structural results, we compare the active sites of topoisomerase IA, IIA and III; and draw their possible similarities and differences in their catalytic mechanisms, particularly between topoisomerase IA and IIA in the role of the metal ions (18,22,23).

## MATERIALS AND METHODS

### Protein expression and purification

Full length (residues 2-934) wild-type and mutant MtbTOP1 enzymes, as well as MtbTOP1-704t (residues 2–704), were expressed in the *Escherichia coli* T7 Express Crystal strain (New England BioLabs) at 22°C following induction with 1 mM IPTG as described (24). The proteins with His_6_-Mocr fusion tag were purified by affinity chromatography using the Ni Sepharose 6 Fast Flow resin (GE Healthcare). For crystallography studies, MtbTOP1-704t was further purified by ssDNA cellulose and gel filtration chromatography following the removal of the fusion tag by TEV protease digestion (24).

### Crystallization

The purified MtbTOP1-704t (77.5 kDa) was concentrated to ∼90 mg/ml (∼1 mM) for crystallization. For the co-crystallization trials with ssDNA, the protein was first mixed with an oligonucleotide in a 1:1 molar ratio and then incubated on ice, typically for 2–3 h before crystallization set-up. To facilitate observation of Mg^2+^ binding in crystal structures, before incubation additional MgCl_2_ stock solution was added to the protein/oligonucleotide mixture in some co-crystallization trials at a final concentration of 2 mM. The crystallization screening and conditions for obtaining the crystals are described in SI. For the preparation of the crystals for X-ray diffraction experiments, they were harvested and transferred to cryoprotectant solution that contains 25% glycerol in addition to crystallization buffer for a few minutes and then cryocooled directly in liquid nitrogen.

### Data collection and structure determination

Single-wavelength diffraction data were collected at 100 K from cryocooled crystals. All data were obtained at the 19-ID beamline of the Structural Biology Center at the Advanced Photon Source at Argonne National Laboratory using the program SBCcollect (25). The intensities of each data set were integrated, scaled, and merged with the HKL-3000 program suite (26). All structures were determined using the molecular replacement method (27). The apo form structure of MtbTOP1-704t (PDB code: 5D5H (24)) was used as the first search template. For the first structure of MtbTOP1-704t/ssDNA complex, the covalent complex of EcTOP1 (*E. coli* topoisomerase I) with ssDNA (PDB code: 3PX7 (28)) turned out to be the best search template. The subsequently refined MtbTOP1-704t/ssDNA structure (PDB code: 6CQI, Table 1) has in turn served as the template for the structural determination of MtbTOP1-704t/ssDNA/Mg complexes. After structural determination, the model rebuilding was performed using the program Coot (29) based on Fourier maps. After several alternative cycles of model building and refinement, the final models were refined using the program phenix.refine (30) (Table 1). There are a few density breaks within the arch-like D2 domain of each structure, particularly in MtbTOP1-704t/ssDNA.

**Table 1.** Data collection and refinement statistics

Data collection	MtbTOP1-704t/MTS2–11	MtbTOP1-704t/MTS2-13/Mg	MtbTOP1-704t (high resolution)	MtbTOP1-704t (second crystal form)
Space group	*P*2_1_	*P*2_1_	*P*2_1_2_1_2_1_	*C*2
Unit Cell dimensions				
*a, b*,*c* (Å)	68.78, 44.98, 129.2	67.63, 44.68, 128.87	63.78, 93.59, 140.6	170.6, 64.42, 169.4
*α, β, γ* (°)	90, 90.60, 90	90, 90.61, 90	90, 90, 90	90, 112.2, 90
Protein MW Da (residue)	77468.3 (703)^a^	77468.3 (703)^a^	77468.3 (703)^a^	77468.3 (703)^a^
DNA MW Da (residue)	3362.2 (11)	3970.6 (13)	NA	NA
Mol or complex (AU)	1	1	1	2
Wavelength (Å)	0.9792	0.9792	0.9792	0.9793
Resolution (Å)	47.0–2.42	37.5–3.00	47.2–2.15	42.3–2.75
Number of unique reflections	29799	15372	46076	45105
Completeness (%)	97.1 (82.4)^b^	97.4 (93.3)^c^	99.5 (99.0)^d^	97.7 (95.7)^e^
Redundancy	4.0 (3.2)^b^	4.6 (2.9)^c^	5.4 (4.8)^d^	5.9 (5.7)^e^
*R*_merge_	0.087 (0.641)^b^	0.132 (0.816)^c^	0.094 (0.800)^d^	0.141 (0.843)^e^
*I*/σ(*I*)	19.5 (1.2)^b^	9.2 (1.1)^c^	17.4 (1.9)^d^	14.3 (1.7)^e^
CC_1/2_	0.82^b^	0.50^c^	0.73^d^	0.52^e^
Wilson *B*-factors (Å^2^)	55.9	63.1	35.2	50.9
***Phasing*** ^f^				
***refinement***				
Resolution	47.0–2.42	37.5–3.00	47.2–2.15	42.3–2.75
No. reflections (work/test)	28222/1484	14613/735	43697/2310	42851/2210
*R*_work_/*R*_free_	0.218/0.272	0.189/0.249	0.168/0.211	0.198/0.243
No.of atoms				
Protein/DNA	5032/202	5289/202	5322/NA	10579/NA
Water/others	18/24	14/9	303/5	47/0
*B*-factors (Å^2^)				
Protein/DNA	95.0/94.5	74.4/79.8	53.1/NA	64.0/NA
Water/Others	69.6/105.2	41.1/83.8	46.8/115.6	40.6/NA
R.m.s deviation				
Bond length (Å)	0.008	0.002	0.007	0.003
Bond angle (°)	1.083	0.418	0.804	0.501
Ramachandran Plot (%) (57)				
Residues in most favored regions,	89.9	87.1	91.8	91.4
in additional allowed regions,	10.1	12.4	8.2	8.4
in generously allowed regions,	0.0	0.2	0.0	0.0
in disallowed region	0.0	0.3	0.0	0.3
**PDB code**	**6CQI**	**6CQ2**	**5UJ1**	**5UJY**

^a^Not including three N-terminal vector-derived residues, SNA.

^b^(Last resolution bin, 2.42–2.46 Å).

^c^(Last resolution bin, 3.00–3.07 Å).

^d^(Last resolution bin, 2.15–2.18 Å).

^e^(Last resolution bin, 2.75–2.80 Å).

^e^ Molecular replacement.

### Enzyme activity assays

The relaxation activity was assayed using supercoiled pBAD/Thio plasmid DNA substrate as described (31). For assay of DNA binding, increasing amounts of wild-type or mutant MtbTOP1 were incubated with 5 pmol of oligonucleotide STS32 (21) labeled with ^32^P on the 5′-end in 10 μl of 20 mM Tris–HCl pH 8.0,100 μg/ml BSA, 18% glycerol, 0.5 mM EDTA at 37°C for 5 min. The reactions were then placed on ice for 5 min before separation of the non-covalent protein–DNA complex and free substrate by electrophoresis in 8% polyacrylamide gel with 0.5X TBE (45 mM Tris-borate pH 8.3, 1 mM EDTA) buffer at 6 V/cm for 4.5 h. The dried gel was analyzed by PhosphorImager. For assay of DNA cleavage activity, the labeled oligonucleotide (0.5 pmol) was incubated with indicated amount of enzyme in 5 μl of 10 mM Tris–HCl (pH 8.0) buffer at 37°C for 30 min. The reactions were terminated by the addition of an equal volume of stop solution (79% formamide, 2 mM NaOH, 0.1 mM EDTA, 0.04% bromophenol blue). After heating at 95°C for 5 min, the oligonucleotide substrates and cleavage products were separated by electrophoresis in a sequencing gel (15% gel for substrate STS32, 20% gel for other oligonucleotide substrates) followed by PhosphorImager analysis.

## RESULTS

### New co-crystals of MtbTOP1-704t with short ssDNA substrates

**Figure 1.**
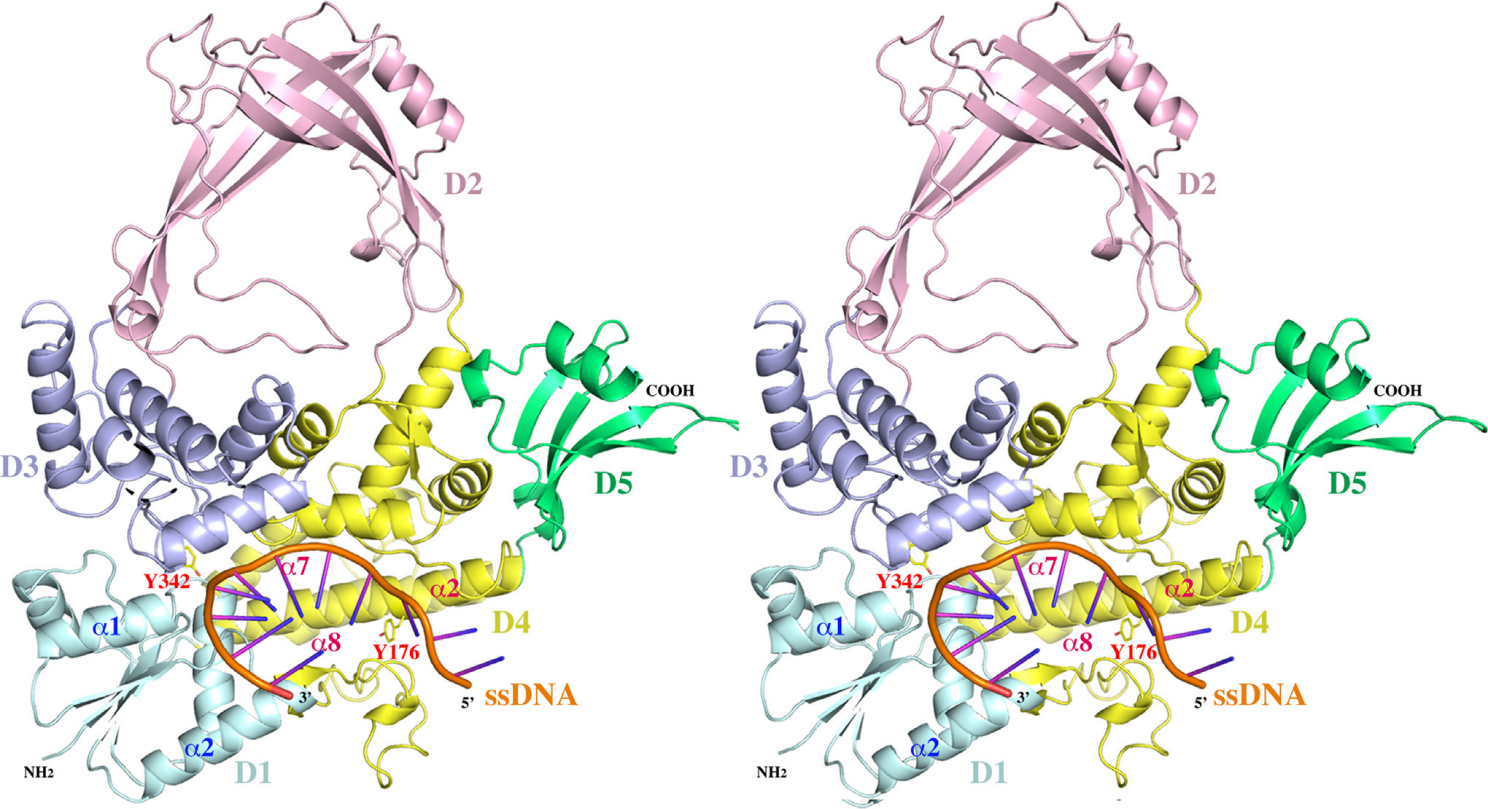
A stereoview of a ribbon diagram of the structure of MtbTOP1-704t/ssDNA complex. The five individual domains, D1–D5, are colored in cyan, pink, blue, yellow and green, respectively. The phosphate backbone of the oligonucleotide substrate is in orange with its polarity labeled. The catalytic residue Y342 is drawn in stick format to highlight its active site position. Y176 is drawn to indicate the relative position between Y342 and the C nucleotide binding pocket (Figure 2B) four bases upstream of scissile phosphate. Only a few secondary structures are labeled for the purpose of discussion. The numberings of these secondary structures are based on individual domains. The conformational changes induced by ssDNA binding can be viewed in SI movies 1 and 2. The program Pymol was used for the preparation of Figures 1–4.

Co-crystallization trials of MtbTOPI-704t (24) were set up with MTS (Mycobacterial Topoisomerase Substrate) oligonucleotides of various lengths with sequences derived from a 32 base long substrate previously identified as having a strong topoisomerase site (STS) (21). Multiple crystal forms were harvested and their X-ray diffraction data were collected. The highest resolution data set (2.42 Å) was obtained from a co-crystal with a short, 11-mer oligonucleotide (MTS2–11, 5′-TTCCGCTTGAC-3′). This crystal structure (MtbTOP1-704t/MTS2–11, PDB ID: 6CQI, Table 1) represents a topoisomerase I/ssDNA complex in the absence of metal ions at the active site, Figure 1.

### Conformational change of MtbTOP1-704t upon ssDNA binding

The MtbTOP1-704t construct includes four N-terminal domains (D1–D4) and one C-terminal domain (D5). D1–D4 form a toroidal assembly found in all topoisomerase IA while D5 represents the Topo_C_Rpt subgroup of bacterial topoisomerase C-terminal repeats (Figure 1 and SI movies 1 and 2) (24). The binding of oligonucleotide MTS2–11 causes a significant conformational change relative to the apo MtbTOP1-704t structure (PDB ID:5D5H), particularly to its N-terminal toroidal assembly. An overall superposition (32) of the holo form structure onto its apo form results in an rmsd (root-mean-square deviation) value of 2.34 Å. The rmsd values from the pairwise alignments of individual domains are 0.94, 0.84, 0.75, 1.86 and 0.63 Å for D1, D2, D3, D4 and D5, respectively.

While D3 contains the catalytic Y342, it exhibits the least conformational change among N-terminal domains upon ssDNA binding. To appreciate the relative domain-domain movements within the toroidal assembly, only the D3 domains from the apo and the holo MtbTOP1-704t structures are used for an alignment calculation. The primary inter-domain re-arrangement can be approximated as a shift of ∼8 Å by D1 with respect to D3 (Figure 1 and SI movie 1 and 2). This movement of D1 from D3, extending the DNA binding groove within D4 to the space between D1 and D3, provides ssDNA access to the active site. D4 itself has the largest conformational change, mostly due to a complete opening of its DNA binding groove by intra-domain helix re-arrangement including rotations, primarily involving α2, α7 and α8 helices (Figure 1 and SI movie 1 and 2). Accompanying the intra-domain rotation within D4 and the inter-domain shift of D1 to D3, the associations of D3 to D1 and D4 are greatly reduced. The interface area between D3 and D1 decreases from 690 to 240 Å^2^ while the interface area between D3 and D4 is reduced from 760 to 510 Å^2^. The ssDNA binding thus contributes to the creation of a gate between D3 and D1 plus D4, which has been proposed to be a critical step for the entry of the T-strand into the interior hole of the toroid in the catalytic mechanism of type IA topoisomerase (33–35).

**Figure 2.**
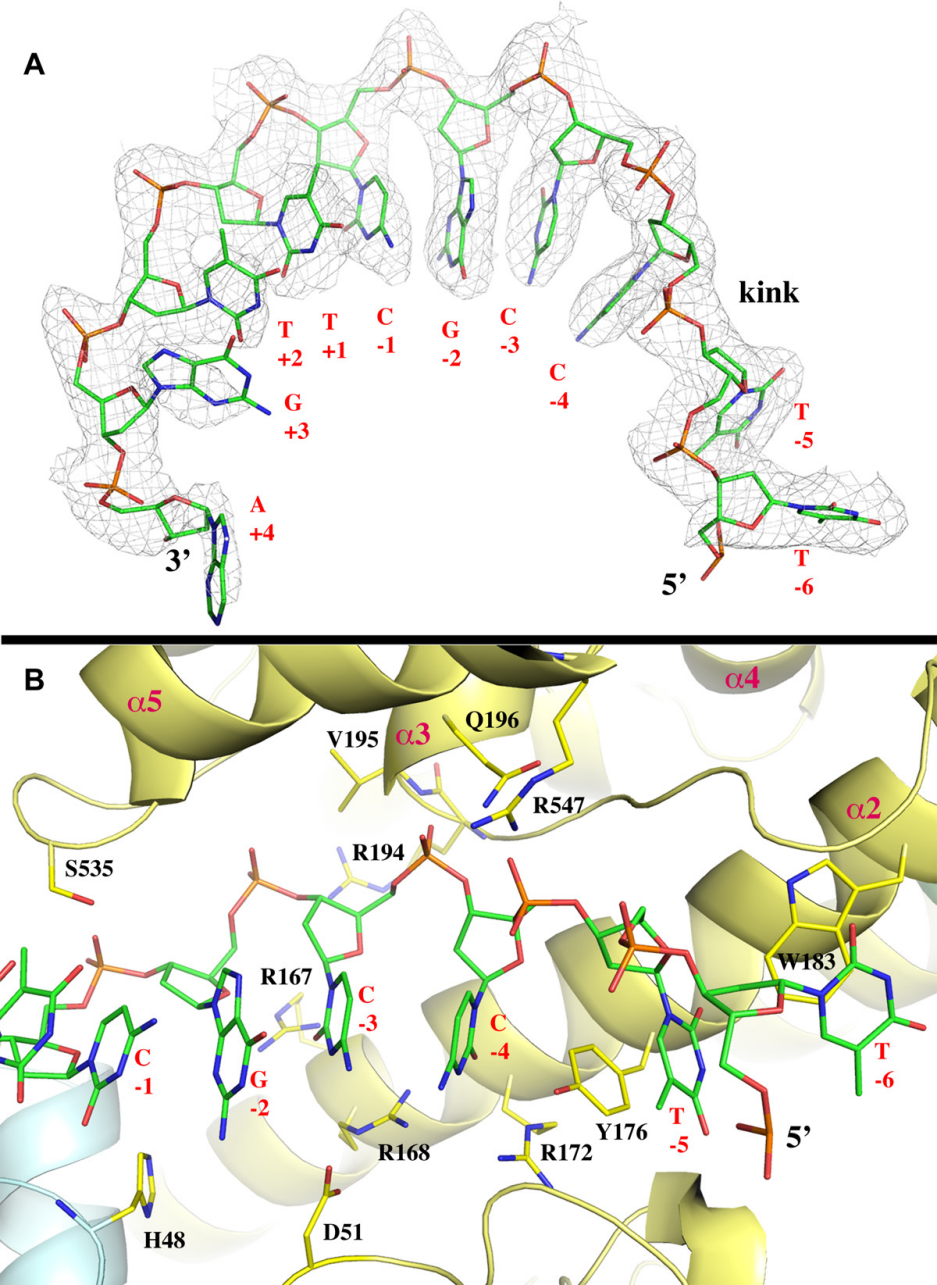
Conformation of ssDNA substrate and its interaction with MtTOP1-704t. (**A**) The ssDNA bound to MtbTOP1-704t resembles a B-DNA conformation, which is interrupted between –4 and –5 positions. The 2Fo-Fc electron density map is drawn in gray mesh and contoured at 1σ level and within 2.1 Å of the ssDNA, which is drawn in stick format. (**B**) Interaction between ssDNA and MtbTOP1-704t including the creation of a ssDNA kink and the C nucleotide binding pocket at –4 position. Several arginine residues involved in the interaction with either the phosphate backbone (R194 and R547) or bases (R167, R168 and R172) are presented. One anchoring site of ssDNA to MtTOP1-704t is located at the phosphate group between –3 and –4 positions. The phosphate group is positioned below the N-terminus of the α3 helix of D4, which has an equivalent charge of about +0.5 unit. In the apo structures of MtbTOP1-704t, a phosphate group or a sulfide group from crystallization buffer is commonly found at this position. The tyrosine Y176 inserts between the bases at –4 and –5 positions, helping create a kink in the base packing of ssDNA. The residue Y176 forms π–π stacking with bases at both –4 and –5 positions. R168 and R172 provide specific interactions to the C nucleotide at –4 position. Both Figure 2A and B were prepared based on the MtTOP1-704t/MTS2–11 structure (PDB code: 6CQI, Table 1).

In addition to the first apo MtbTOP1-704t structure reported earlier (24), more apo MtbTOP1-704t structures (e.g. PDB ID:5UJ1 and 5UJY, Table 1) have been determined from crystals grown under different conditions. No significant conformational change has been observed among these structures even though they have different crystal symmetries. This indicates that the N-terminal toroidal assembly of the enzyme is quite stable by itself. The conformational change induced by ssDNA binding in the structure of MtbTOP1-704t/MTS2–11 is comparable to that observed in the EcTOP1 covalent complex (28). The rmsd value from an overall alignment of the two structures is only 1.69 Å, significantly less than the rmsd value from the superposition of the *apo* to *holo* form of MtbTOP1-704t as described above. Since ssDNA is intact in the complex structure (Figure 2A), this suggests that this MtbTOP1-704t/MTS2–11 structure may correspond to a pre-catalytic state poised for DNA cleavage in the absence of Mg^2+^ ion as elaborated later. This structure may also resemble the post-ligation state.

### Conformation of ssDNA substrate and its kink

**Figure 3.**
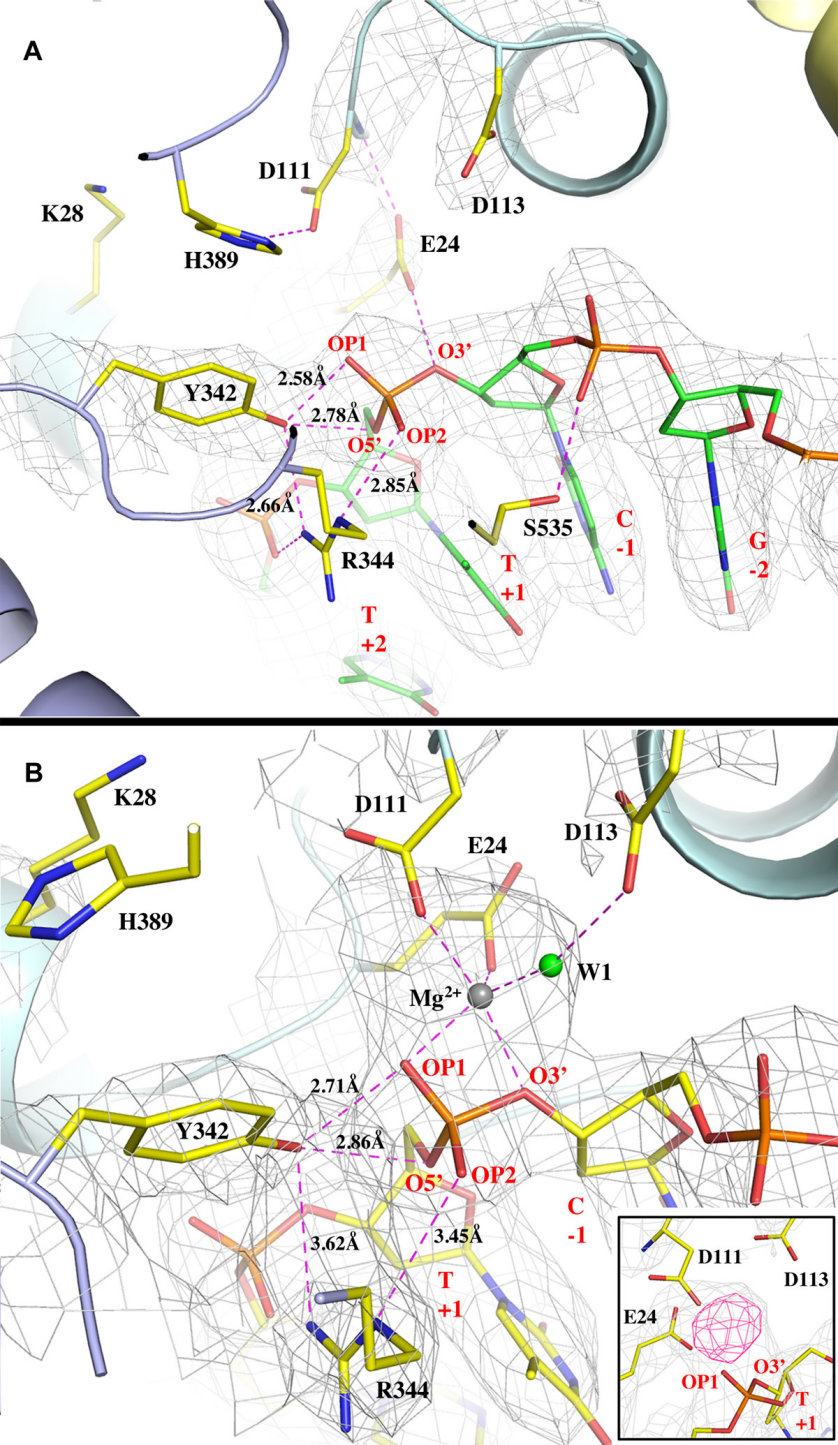
Active sites in the absence and in the presence of Mg^2+^ ion. (**A**) The active site interaction pattern between MtTOP1-704t and ssDNA in the absence of Mg^2+^ ion. The key residues from MtbTOP1-704t and ssDNA are drawn in stick format. All hydrogen bonds and salt bridges are drawn in magenta dash lines with some bond distances labeled. The distance between the phenolic oxygen of Y342 to the scissile phosphorus atom is 2.90 Å. The residue K28, as discussed in text, is too far away from the scissile phosphate. The 2*F*_o_ – *F*_c_ electron density map is drawn in grey mesh and contoured at 1σ level and within 2.1 Å of the ssDNA and the residues of E24, D111, D113 and Y342. (**B**) The active site interaction pattern between MtbTOP1-704t and ssDNA in the presence of Mg^2+^ ion. The MtbTOP1-704t complex shown in this figure is slightly rotated along the horizontal axis with respect to that shown in A for better view of the Mg^2+^ ion binding site. In addition to hydrogen bonds and salt bridges, all Mg–O bonds are also drawn in magenta dash lines. The distance between the phenolic oxygen of Y342 to the scissile phosphorus atom is 3.13 Å. The 2*F*_o_ – *F*_c_ electron density map is drawn in grey mesh and contoured at 1σ level and within 2.1 Å of the ssDNA, the residues of E24, D111, D113 and Y342, the metal ion, and water molecules. The insert figure displays simulated annealing omit maps of the metal-binding site, 2*F*_o_ – *F*_c_ map in grey at contour level of 1σ, and *F*_o_ – *F*_c_ map in pink at contour level of 5σ. The simulated annealing refinement with a starting temperature of 5000 K was performed after omitting Mg^2+^ and W1. Figure A was prepared based on the MtTOP1-704t/MTS2–11 structure (PDB code: 6CQI, Table 1). Figure B was prepared based on the MtTOP1-704t/MTS2-13/Mg structure (PDB code: 6CQ2, Table 1).

The ssDNA MTS2–11 is intact in the structure (Figure 2A). Its sugar phosphate backbone is mostly buried along the bottom of the DNA-binding groove from D4 to the edge of the D1-D3 interface in a 5′ to 3′ direction (Figure 1). The bases of the seven nucleotides from positions –4 to +3 resemble the stacking conformation of one B-DNA strand as described earlier for DNA bound to *E. coli* topoisomerase III (EcTOP3) (36) and topoisomerase I (EcTOP1) (37). This conformation is interrupted between the –4 and –5 positions with a backbone kink and insertion of a tyrosine side chain from the enzyme (Y176) between the two bases (Figure 2B). The kink contributes to the recognition of cytosine at the –4 position relative to the scissile phosphate for all preferred bacterial topoisomerase I cleavage sites (28,31,38). Aside from the interactions with the –4 cytosine base involving R168, R172 and Y176, there are additional enzyme–ssDNA interactions, including a series of arginine–phosphate salt bridges mediated by R194, R344 and R547 (Figures 2B and 3). The specific interaction with the cytosine base at the –4 position as seen in the EcTOP1 covalent complex structure also provided additional support for assigning this structure as corresponding to the pre-transition state in the reaction pathway that would lead to the formation of the covalent complex.

### Active-site conformation in the absence of Mg^2+^ binding

The conformational change upon ssDNA binding not only helps to create the gate for the passing of the T-strand, it also brings catalytic residues together to form an active site. The active site configuration of MtbTOP1-704t/MTS2–11, in the absence of Mg^2+^ ion, is similar to that of the covalent complex of EcTOP1/ssDNA(28) and EcTOP3/ssDNA (36) complexes, but with new features (Figure 3A). The catalytic tyrosine Y342 makes two hydrogen bonds from the top to the scissile phosphate, including one to its bridging 5′-oxygen atom and one to a non-bridging O atom (Figure 3A). The distance between the hydroxyl group of Y342 to the phosphorous of the scissile phosphate is 2.90 Å. The scissile phosphate is being positioned on one side by R344 through an arginine-phosphate salt bridge, and on the other side by E24 through a hydrogen bond from its bridging 3′-oxygen atom (Figure 3A). E24 also forms a hydrogen bond with the amide group of D111, bridging the catalytic residue to the scissile phosphate.

The MtbTOP1-704t/MTS2–11 structure does not have a positively-charged lysine side chain interacting with the DNA at the active site. In contrast, lysine K8 in the EcTOP3/ssDNA complex interacts with the putative scissile phosphate. The positively charged K8 was believed to help stabilize the negative charge on non-bridging oxygens of the pentavalent transition state (36). However, in MtbTOP1 as well as in EcTOP1, this lysine residue is not conserved, being replaced by a serine in the bacterial topoisomerase I sequence (39). A nearby lysine K28 in MtbTOP1 (equivalent to K13 in EcTOP1) is >6.8 Å away from the scissile phosphate based on measurement of the distances between the K28 Nζ atom to the oxygen atoms of the phosphate (Figure 3A). The absence of an EcTOP3 K8 equivalent in MtbTOP1 and EcTOP1 active site structures (Supplementary Figure S1) implies some difference between the catalytic mechanisms of bacterial topoisomerase I and topoisomerase III, as suggested earlier (28,40). The side chain of D111 is quite mobile and without well-defined electron density. D111 can potentially make a hydrogen bond with an invariant H389 on the long loop between α3 and α4 helices of D3. H389 also can potentially form a π-π stacking with Y342 in this MtbTOP1-704t/MTS2–11 complex. It is noted that the α3_α4 loop of D3 is very flexible, allowing the position and conformation of the strictly-conserved histidine to be highly variable from structure to structure. Similar observations have been made for the covalent EcTOP1 complex (28) as well as in the open and the mutant forms of EcTOP3, in which the histidine forms a nearly parallel displaced π-π stacking with the catalytic tyrosine (36,41). However, in the closed and the intermediate forms of EcTOP3, there is no interaction between these two residues. The flexible α3_α4 loop of D3 and its conserved histidine could play a potential role in charge relay for DNA cleavage and religation (28,42). The direct π-π interaction with the catalytic tyrosine may also allow the invariant histidine to assist in stabilization of the transition state for DNA cleavage in the absence of Mg^2+^.

### Structure of active site with bound Mg^2+^ ion

Mg^2+^ plays an essential role in DNA religation by type IA topoisomerase (17,21,43). However, divalent metal ions have never been observed in any co-crystal structures of the topoisomerase IA-DNA complex. After extensive screening of MtbTOP1-704t/ssDNA crystals grown under different conditions, one crystal structure of MtbTOP1-704t in complex with a 13-mer oligonucleotide, MTS2-13 (PDB ID:6CQ2, Table 1) reveals the first metal-binding mode at the enzyme active site (Figure 3B). Compared to the structure of MtbTOP1-704t/ssDNA in the absence of metal ion (Figure 3A), the extra electron densities in an approximately globular shape between the scissile phosphate and the carboxyl groups of E24 and D111 strongly indicate a site of bound Mg^2+^ ion (Figure 3B). One water molecule, W1, was assigned to coordinate the metal ion, forming a distorted octahedral geometry with one ligand missing. It should be mentioned that the position of the side chain of D111 is not well-defined in electron density maps, suggesting that this residue can adopt multiple positions, with one of the positions engaged in metal binding. After applying Mg-O bond distance restraints (2.10 ± 0.20 Å) to the metal-binding site in subsequent structural refinements, the distorted octahedral geometry is stable and the model fits the electron densities well. Interestingly, the water W1 can form a hydrogen bond with another TOPRIM acidic residue, D113 (Figure 3B), proposed previously (19,21) to interact with Mg^2+^.

After obtaining this MtbTOP1-704t/MTS2-13/Mg structure, a second structure with a metal ion bound at the same site was determined after screening of crystals formed in different conditions, particularly in conditions with added MgCl_2_ in the crystallization buffers (see experimental details in SI Methods). In the co-crystal, harvested from a condition with added MgCl_2_, there are two MtbTOP1-704t/ssDNA/Mg complexes in one asymmetric unit. Both complexes show the aforementioned Mg^2+^ binding site, but with partial occupancies of the metal ion (data not shown). The addition of Mg^2+^ in the crystallization buffer did not simply increase Mg^2+^ binding, at least under the buffer condition used (SI Methods).

To evaluate any conformational change possibly induced by Mg^2+^ binding, two structures, MtbTOP1-704t/MTS2–11 and MtbTOP1-704t/MTS2-13/Mg were superimposed. The resulted small rmsd value of 0.69 Å from the alignment indicates no overall structural impact upon Mg^2+^ binding. A close examination of their active sites also found no major changes aside from the side chain of R344, which moved away >0.6 Å from Y342 and the scissile phosphate. Considering that the ssDNA is intact and that the distance between Y342 hydroxyl and the phosphorous of the scissile phosphate is 3.13 Å, we propose that this MtbTOP1-704t/MTS2-13/Mg structure may represent an enzyme-ssDNA complex following DNA religation in the presence of Mg^2+^, as further elaborated later. The presence of Mg^2+^ would shift the cleavage-religation equilibrium from DNA cleavage toward DNA religation (43).

### Comparison of metal-binding sites between type IA and IIA topoisomerase complexed with DNA

**Figure 4.**
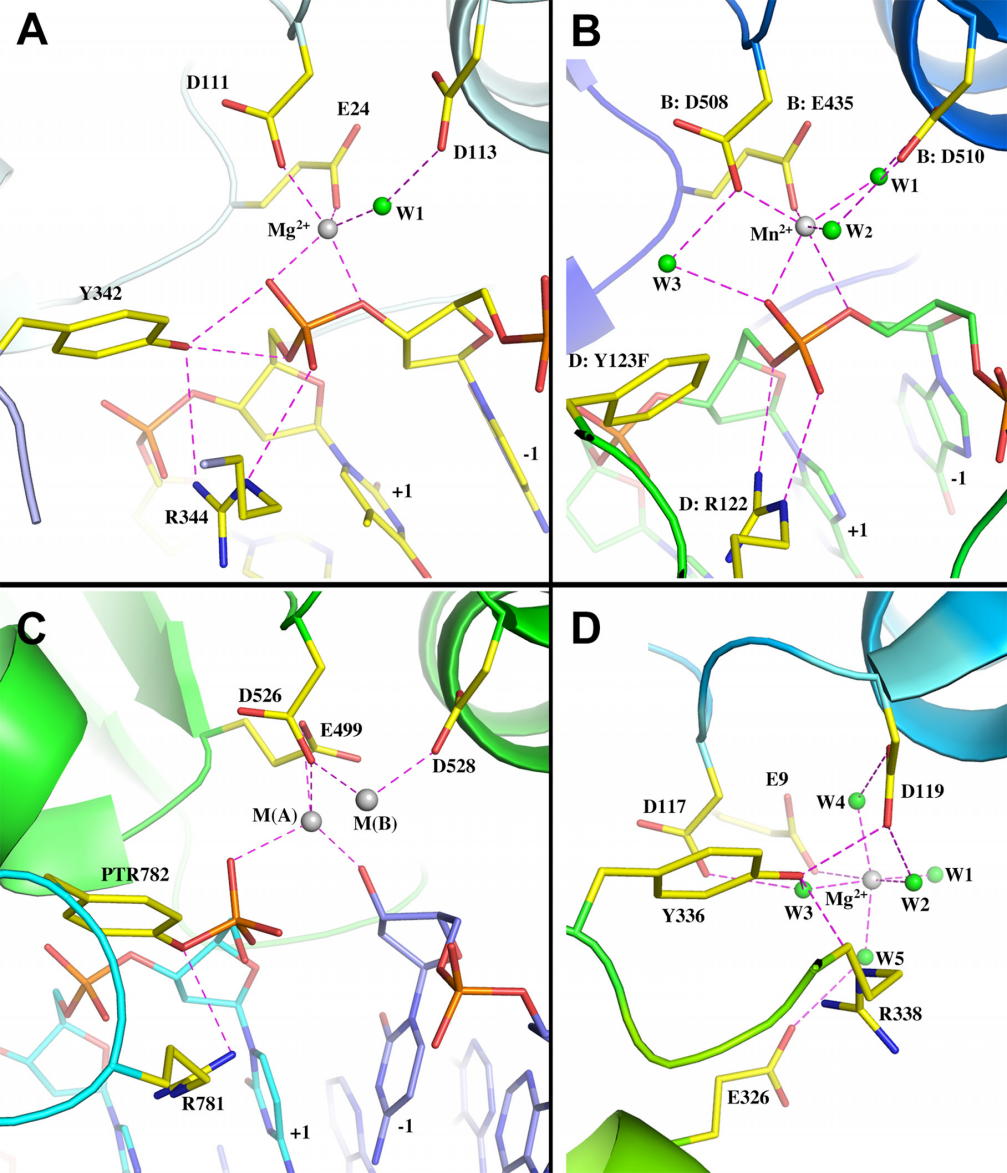
Comparison of metal-binding sites of type IA and IIA topoisomerases. (**A**) Ribbon diagram of Mg^2+^ binding site of MtTOP1-704t/MTS2-13/Mg (PDB code: 6CQ2). (**B**) Ribbon diagram of the Mn^2+^ binding site of *S. aureus* type IIA topoisomerase complex with inhibitor and DNA (PDB code: 2XCS). Residues are labeled based on the numberings in PDB file. Waters are renumbered for convenience. (**C**) Ribbon diagram of the *S. cerevisiae* type IIA topoisomerase covalent complex with two Zn^2+^ binding sites (PDB code: 3L4K). Residues are labeled based on the numberings in PDB file. (**D**) Ribbon diagram of the active site of human topoisomerase IIIα (PDB code: 5GVC). Residues are labeled based on the numberings in PDB file. Waters are renumbered for convenience.

The first observation of Mg^2+^ binding at the active site of topoisomerase IA as described above supports the proposed roles of the TOPRIM acidic residues in metal binding (19). We have compared the observed metal binding at active sites in type IA topoisomerases to those revealed in type IIA topoisomerases and in human topoisomerase III (Figure 4).

Among the type IIA topoisomerases that have been more extensively studied, one metal-binding site and two metal-binding sites have variously been reported between different structures. Two different catalytic mechanisms have been proposed to account for the different observations: (a) a mechanism with a single catalytic metal, and a second non-catalytic metal that are simultaneously occupied; and (b) a moving metal mechanism involving only one metal ion. The type IIA topoisomerase from *S. aureus* in complex with DNA, an inhibitor, and Mn^2+^ ion is of the highest structural resolution (2.1 Å) among all known type IIA topoisomerase structures in complex with DNA (44). The metal-binding site, including all coordinated water molecules, is well-defined in electron density maps. The architectures of the metal-binding sites of type IA (Figure 4A) and type IIA (Figure 4B) topoisomerases are nearly parallel, excepting a few details of water coordination. In the MtbTOP1-704t/MTS2-13/Mg structure described above, the water W1 (Figure 4A) was largely from an assignment based on metal coordination geometry due to the structural resolution limit. Two Zn^2+^ metal ions can be observed in the structure obtained for *S. cerevisiae* topoisomerase II (23). The catalytic metal M(A) in this structure (Figure 4C) is believed to participate directly in transition state stabilization and in promoting the leaving and attacking of the O3′ of the scissile phosphate during DNA cleavage and religation, while the metal M(B) is proposed to anchor the DNA (18,22,23). Though certain details between the proposed mechanisms for type IA and type IIA topoisomerases differ, their overall similarity lends support to a long-time proposal that type IA and type IIA topoisomerases may have similar catalytic mechanisms, at least for DNA religation (15).

### Comparison of metal-binding sites in type IA topoisomerases in presence/absence of DNA

Metal binding has never been observed in prior bacterial type IA topoisomerase structures. During the study of MtbTOP1-704t, when crystals from many different conditions were screened, no metal binding had been observed in any apo form structures, even as we gradually improved the resolution limit from earlier-reported 2.52 to 2.15 Å (Table 1). Human topoisomerase IIIα is the first type IA structure (45) to show a metal-binding site in the absence of bound DNA (Figure 4D). Structural alignments of the human topoisomerase IIIα to apo MtbTOP1-704t (PDB code: 5UJ1, Table 1) and MtbTOP1-704t/MTS2-13/Mg (PDB code: 6CQ2, Table 1) yield rmsd values of 2.39 and 2.75 Å, respectively. It indicates that the metal-bound topoisomerase III is closer to the apo form of MtbTOP1-704t than to its holo form. This is likely due to the absence of bound DNA in the human topoisomerase IIIα structure.

An examination of the metal-binding site of the human topoisomerase IIIα and a comparison to the DNA-dependent metal-binding site in MtbTOP1 (Figure 4) provide the following information. (i) The highly conserved glutamic acid (E24 in Figure 4A, E9 in Figure 4D) from D1 (TOPRIM domain) directly interacts with metal ion in the presence/absence of DNA. (ii) The two TOPRIM aspartate residues (D111 and D113 in Figure 4A, D117 and D119 in Figure 4D) can interact either directly or indirectly through water bridge(s) with the metal ion. The side chains of these two acidic residues appear to be relatively flexible, implying their potential for multiple conformations. (iii) The metal-binding site mediated by the acidic residues mentioned above needs to be stabilized by the presence of a bound DNA (Figure 4A and B). This requirement indicates that the metal-bound topoisomerase III structure may represent an intermediate state between an apo and a holo (DNA-binding) state. We believe that a similar intermediate state between apo and holo can also exist in MtbTOP1, which has not yet been selectively crystallized.

### Mapping of exact cleavage site on single-stranded oligonucleotide substrates

**Figure 5.**
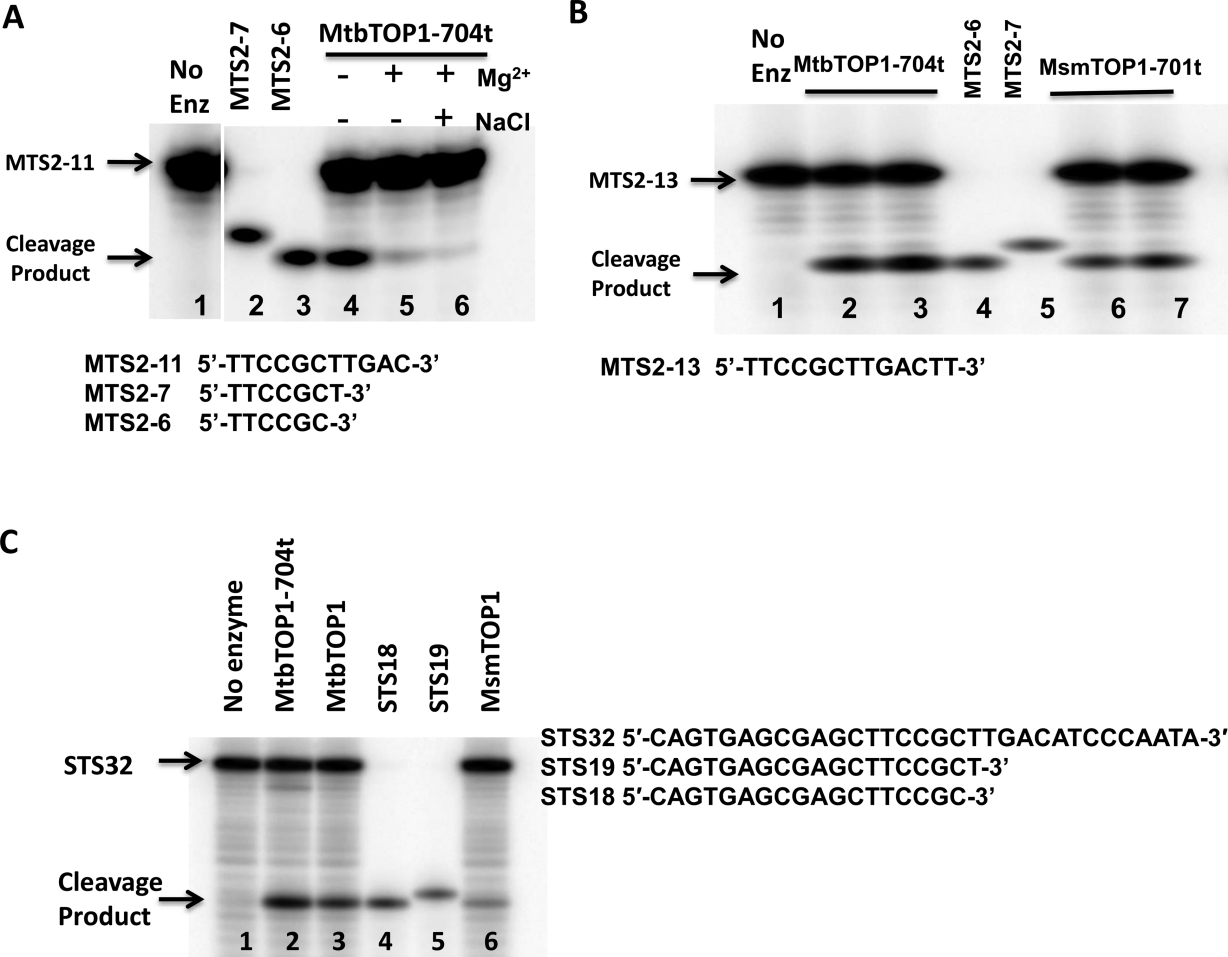
Mapping of exact cleavage site on single-stranded oligonucleotide substrates. (**A**) Cleavage and religation of MTS2–11 substrate by MtbTOP1-704t. Lane 1: MTS2–11 substrate with no enzyme added. Lane 2: MTS2-7 oligo standard. Lane 3: MTS2-6 oligo standard. Lane 4: cleavage by 1 pmol MtbTOP1-704t. Lane 5: cleavage by 1pmol MtbTOP1-704t with 0.5 mM Mg^2+^ added for DNA religation. Lane 6: cleavage by 1pmol MtbTOP1-704t followed by addition of 0.5 mM Mg^2+^ and 1 M NaCl to dissociate the enzyme following DNA religation. (**B**) Cleavage of MTS2-13 by MtbTOP1-704t and MsmTOP1-701t. Lane 1: MTS2-13 substrate with no enzyme added. Lanes 2, 3: 1 and 2 pmol of MtbTOP1-704t added. Lanes 4: MTS2-6 oligo standard. Lane 5: MTS2-7 oligo standard. Lanes 6, 7: 1 and 2 pmol of MsmTOP1-701t added. (**C**) Cleavage product with STS32 oligonucleotide substrate. Lane 1: STS32 substrate with no enzyme added. Lanes 2, 3 and 6: 1 pmol MtbTOP1-704t, MtbTOP1 and MsmTOP1 added respectively. Lanes 4: STS18 oligo standard. Lane 5: STS19 oligo standard.

In each of the crystal structures of the MtbTOP1/DNA complex reported here, there is a DNA phosphate positioned at a distance (∼3.0 Å) from catalytic Y342. In order to verify that the phosphate is indeed the scissile phosphate for the cleavage of bound oligonucleotides (MTS2–11 or MTS2-13), we performed PAGE analysis of the cleavage product, using shorter 5′-end labeled oligonucleotides for comparison (Figure 5A and B). These experimental results are consistent with the structural observations. We also confirmed that both MtbTOP1 and *M. smegmatis* topoisomerase I (MsmTOP1) cleave at the same site in oligonucleotide STS32, a 32-base long ssDNA oligonucleotide from which the sequences of the shorter oligonucleotide substrates were derived (Figure 5C). This cleavage position is shifted by one nucleotide toward the 5′-end from the position expected based on the previous report (21). This could be due to differences in enzyme preparation, cleavage reaction conditions, or cleavage site mapping protocols (46). Additionally, the identification of the scissile phosphate further confirms the recognition of a cytosine at the –4 position relative to the scissile phosphate, as discussed earlier (28,31,38).

### Requirement of the strictly conserved Glu24 for DNA cleavage

The glutamate E24 and the two aspartates present as DxD (D111 and D113) are strictly conserved acidic residues that have been found to be critical in the catalysis of type IA and IIA topoisomerases, as well as other nucleotidyl transfer enzymes that require Mg^2+^ for their activities (19). Their contribution to the active site as described above supports their roles in the catalytic activities of these topoisomerases. Despite the weak electron densities for the side chains of D111 and D113 in the current structural models, the roles of these two catalytic residues in Mg^2+^ coordination in the active site observed here are entirely consistent with previous mutational analysis of the corresponding residues in *E. coli* and *M. smegmatis* topoisomerase I (20,21,47). We have attempted to construct a D111N mutant MtbTOP1 clone, but only clones with additional mutations in the coding sequence could be isolated in *E. coli*, likely due to the lethality associated with inhibition of DNA rejoining.

The residue E24 in MtbTOP1 and the residue E9 in EcTOP1 are equivalent in both sequence and structure. Site-directed mutagenesis of EcTOP1 has shown that alanine substitution at E9 abolishes DNA relaxation activity, and the E9A mutant could not cleave DNA (48,49). A role for this conserved glutamate in acid-base catalysis of DNA cleavage and rejoining has been proposed, with the glutamate side chain providing the proton for the 3′-OH leaving group during DNA cleavage (42), and enhancing the nucleophilic properties of the 3′-OH during DNA religation (28). In the structure with MTS2–11 in the absence of divalent ions (Figure 3A), E24 of MtbTOP1 is well positioned to donate a proton to the 3′-OH leaving group. Partly due to the placement of this strictly conserved glutamate, the MtbTOP1-704t/MTS2–11 structure is seen as the probable structure prior to the DNA cleavage step. In the structure with MTS2-13 in the presence of Mg^2+^ (Figure 3B), E24 is critical in forming and positioning the binding site for Mg^2+^, which can then increase the nucleophilicity of the 3′-OH during DNA religation. The position of E24 in the MtbTOP1-704t/MTS2-13/Mg structure also supports the assignment of this structure as the likely structure following DNA religation.

The critical functions of the strictly conserved glutamate E24 may be assisted by charge relay involving D111 and H389, with water molecules in the hydrogen-bonding network at the active site being potential participants in acid-base catalysis. D111 and H365 in EcTOP1 are well positioned for the proposed charge relay (42,50). The sequence of charge relay during DNA cleavage would involve donation of a proton from the positively charged invariant histidine to the nearby aspartate, which then relays the charge to the strictly conserved glutamate so that it can be protonated at physiological pH to act as a general acid for neutralizing the negative charge on the 3′-hydroxyl leaving group. The bell-shaped pH dependence of EcTOP1 catalysis is modified by H365 mutations (42), consistent with the role of this conserved histidine in optimal catalysis at physiological pH (42). A nearby water molecule can potentially replace the role of the histidine for donating a proton if the histidine is mutated to another residue. However, the role of EcTOP1 glutamate E9 as a general acid during DNA cleavage is inconsistent with the reported retention of DNA cleavage activity for the EcTOP1 E9Q mutant (49).

**Figure 6.**
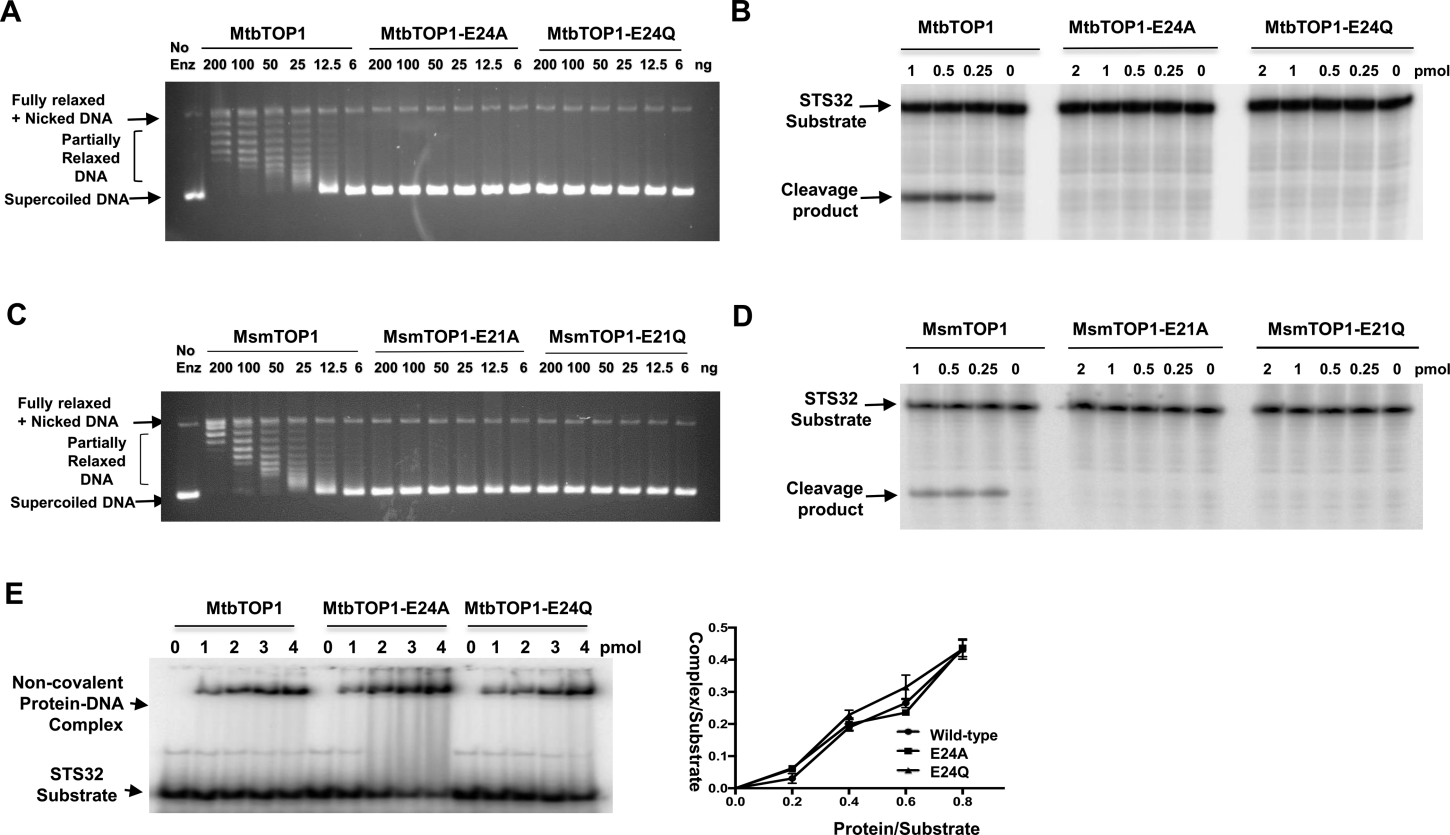
Substitutions of invariant glutamate prevent relaxation of supercoiled DNA and ssDNA cleavage by mycobacterial topoisomerase I without affecting non-covalent DNA binding. (**A**) Relaxation assay: Agarose gel electrophoresis of products from incubation of supercoiled DNA with wild-type and E24A, E24Q mutant MtbTOP1 at 37°C for 30 min. (**B**) ssDNA cleavage assay: STS32 oligonucleotide labeled with ^32^P at the 5′-end was incubated with wild-type and mutant MtbTOP1. (**C**) Relaxation assay of wild-type and E21A, E21Q mutant MsmTOP1. (**D**) ssDNA cleavage of wild-type and mutant MsmTOP1. (**E**) ssDNA binding assay of wild-type and mutant MtbTOP1: Electrophoretic mobility shift assay for formation of non-covalent complex between labeled STS32 oligonucleotide and enzyme. The graph shows average and standard means from results of three experiments.

In order to clarify the catalytic role of this critical conserved glutamic acid residue, we mutated E24 of MtbTOP1 to alanine or glutamine. Both the MtbTOP1-E24A and MtbTOP1-E24Q mutants had null relaxation activity as well as no observable DNA cleavage activity (Figure 6A and B). Similar results were obtained for the MsmTOP1 E21A and E21Q mutants (Figure 6C and D). Gel shift assays of non-covalent binding to the oligonucleotide substrate showed that the binding of the MtbTOP1-E24A and MtbTOP1-E24Q mutant proteins remain unaffected prior to the DNA cleavage step (Figure 6E). To confirm that the conserved glutamate in EcTOP1 functions in the same capacity, we also showed that the EcTOP1-E9Q mutant protein had no relaxation activity and no detectable cleavage activity when tested with substrate ssDNA under our experimental conditions (Supplementary Figure S2). In the report of the previous study of the EcTOP1-E9Q mutation, it was stated that: ‘Further confirmation of the presence of the intended mutation in each of the mutant plasmid was carried out by DNA sequencing’ (49). If the EcTOP1 gene for the mutant clone in this previous study has not been sequenced in entirety, it cannot be ruled out that there may be other substitutions to account for the differences in cleavage activity observed.

## DISCUSSION

In a model of DNA cleavage proposed for type IIA topoisomerases (23), a divalent metal ion and a conserved arginine interact with the scissile phosphate to stabilize the transition state while a second metal ion interacts with the -1 phosphate to position the scissile phosphate in the active site for cleavage by the tyrosine nucleophile (Figure 4C). Alternatively, a moving metal mechanism involving only one metal ion has been proposed based on two *S. aureus* type IIA gyrase structures (44), in which a metal-binding site is in different positions at the active site, 2.8 Å apart between a pre-cleavage structure (PDB 2XCS, Figure 4B) to a structure with DNA cleaved (PDB 2XCT).

**Figure 7.**
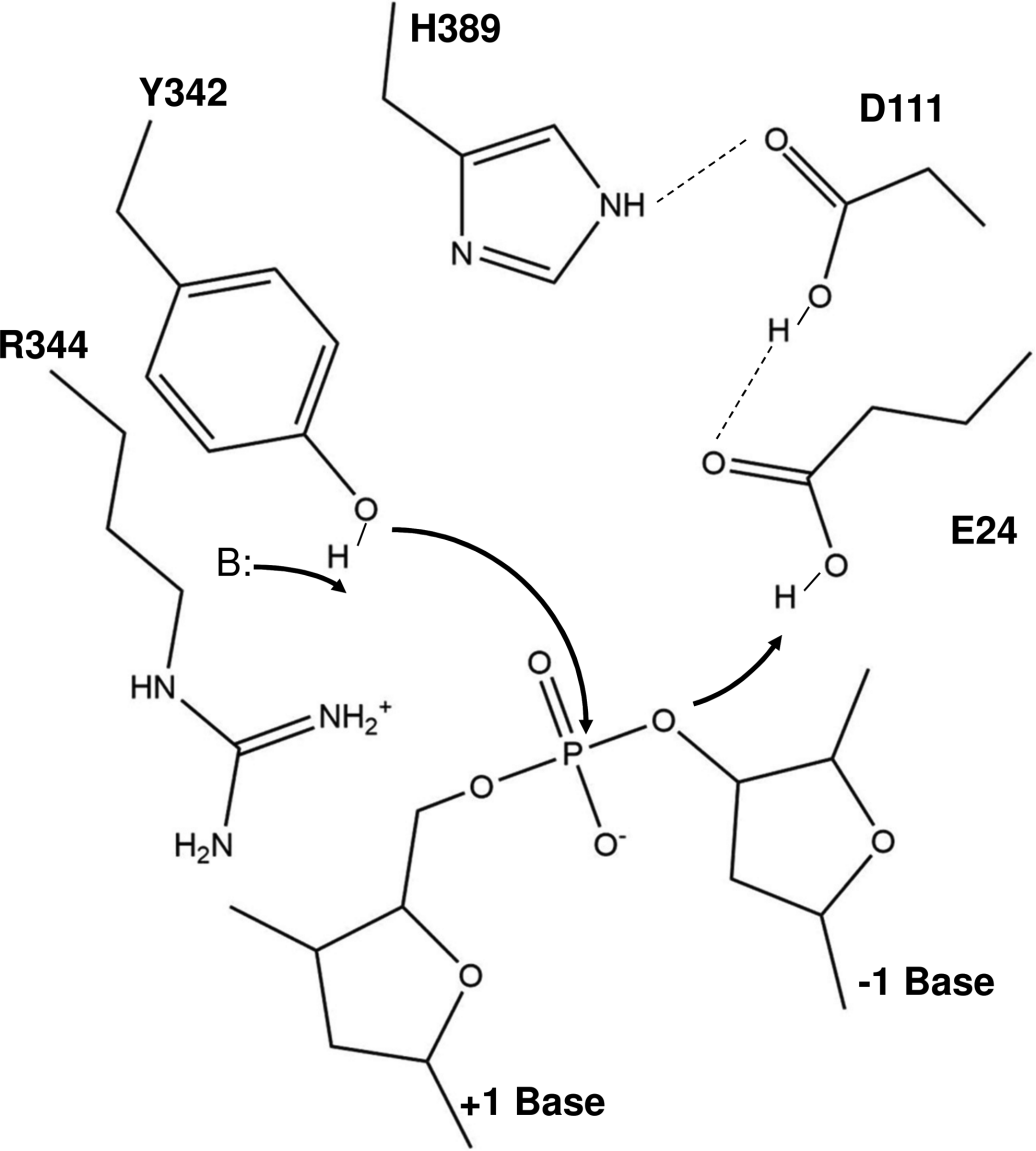
Proposed scheme for DNA cleavage in the absence of divalent ions. The scheme is based on the structure of the MtbTOP1-704t/ssDNA complex (PDB 6CQI). The active site tyrosine Y342 hydroxyl group is positioned for attack of the scissile phosphate through the network of non-covalent interactions extending from the vicinity of Y342 to Y176 shown in Figures 1 and 2. A water nearby that is invisible in the PDB structure is required to act as general base (B:) to activate the hydroxyl nucleophile. The positive charge of the Arg344 side chain plays a critical role in stabilizing the negative charge on the hydroxyl nucleophile and the transition state. The E24 side chain interacts directly as general acid with the DNA 3′-hydroxyl leaving group to provide a proton that can be extracted from the positively charged H389 side chain via proton relay through the D111 side chain.

We have noticed that in the MtbTOP1-704t/MTS2-13/Mg structure there were additional smeared electron densities between D111 and Y342 (not shown in figures). Due to the limit of structural resolution, it is uncertain if there is a second metal-binding site at the active site based on this MtbTOP1-704t/MTS2-13/Mg structure. Because of this uncertainty, we refrain from proposing a comprehensive catalytic mechanism for bacterial topoisomerase I that specifies a single metal ion or two metal ions that would be required for DNA religation. However, the active site features observed in the newly available structures, in combination with the functional data, do shed light on some catalytic steps that should apply to all bacterial topoisomerase I enzymes due to the very high degree of conservation of the active site residues and structures within this group. For metal-independent DNA cleavage (proposed scheme shown in Figure 7), a water molecule in the vicinity of the active site tyrosine Y342 may act as a general base to extract a proton from the Y342 hydroxyl side chain, initiating nucleophilic attack. The strictly conserved arginine (R344 in MtbTOP1) then interacts with both the deprotonated tyrosine hydroxyl oxygen and the scissile phosphate to stabilize the negative charge of the phosphorane transition state. Secondly, E24 could act as general acid to protonate the 3′-O leaving group. D111 and H389 are in place for charge relay to assist E24 (Figure 3A). This scheme proposed for DNA cleavage is supported by the previously observed requirement of Mg^2+^ for DNA cleavage by EcTOP1 mutants where the conserved arginine (R321) is altered to an aromatic residue that can interact with the active site tyrosine through hydrophobic interaction, but cannot provide any electrostatic interaction (51). Additionally, the stacking interaction between the invariant histidine (H389 in MtbTOP1) and the catalytic tyrosine may also help to stabilize the DNA cleavage transition state in the absence of Mg^2+^. While we cannot rule out additional conformational change has to take place following the formation of the holo structure shown in Figure 3A prior to DNA cleavage taking place, it is possible that the water molecule needed as general base is not in the prerequisite position in this holo structure to extract the proton from the active site tyrosine side chain in order to initiate DNA cleavage. This may explain why DNA cleavage does not take place in the crystal. It is also noted that while D111 and D113 side chains in the structure seen here are less ordered, the N111 and D113 side chains in the covalent complex of EcTOP1 are both ordered. The precise positioning of these residues may be needed for DNA cleavage to take place.

Because type IA topoisomerases break and rejoin a single-strand of DNA that is not base-paired to its complementary strand, Watson-Crick base pairing is not available to help align the 3′-OH and 5′-phoshoryl group linked to the tyrosine in the covalent intermediate for rejoining. Therefore, we believe that in the metal-dependent ligation, metal ion(s) may be required for these DNA ends to be joined by a phosphodiester linkage in the DNA religation step. In Figure 3B, the observed Mg^2+^ ion can help activate the 3′-hydroxyl group as a nucleophile while E24, D111 and D113 help to position the metal ion and consequently the 3′-hydroxyl group at a required distance from the phosphotyrosine linkage. Based on this study, it is not clear if there is a second Mg^2+^ ion present during religation, which could be responsible for positioning the 5′-phosphoryl tyrosine and stabilization of the transition state for DNA rejoining. One possible scenario is that there is such a second metal-binding site, which dissociates after DNA rejoining is done. This scenario might explain why the second metal-binding site is not readily observed. It is worthwhile to emphasize that the positive charge of the conserved arginine (R344 in MtbTOP1) interacting with the active site tyrosine leaving group is essential for DNA rejoining. DNA cleavage intermediates accumulate *in vivo* when this arginine in EcTOP1 (R321) is mutated to a hydrophobic amino acid (51). The presence of Mg^2+^ ions cannot compensate for the absence of the arginine positive charge necessary for DNA rejoining. Therefore even though the second metal ion may be in a position to stabilize the negative charge of the tyrosine hydroxyl leaving group as expected for the canonical two-metal ion mechanism, the positive charge of this arginine is still required for interacting with the tyrosine hydroxyl negative charge during DNA rejoining.

We understand that the roles proposed for the divalent ion described above differ from the classic two-metal ion mechanism, in which both metal ions interact with the transition state, with one assisting in the activation of the nucleophile while the other stabilizes the leaving group (52). There are also significant differences from the two-metal ion mechanism proposed for type IIA topoisomerase (23), with one metal ion assisting the acid-base catalysis by interacting with the transition state and the second metal ion interacting with an adjacent phosphate to anchor the substrate DNA. Moreover, we also cannot disregard the ‘one moving metal’ mechanism (44). Particularly, this is because very similar metal-binding sites have been observed in the crystal structures of both type IA and type IIA topoisomerases (Figure 4).

The preferred DNA cleavage sites of MtbTOP1 have a cytosine base at the –4 position upstream of the scissile phosphate (31). This cleavage site preference holds true for all bacterial topoisomerase I, but not for topoisomerase III. We propose that the arginines (R194 and R547) and the N-terminus of the α3 helix of D4 interact with the phosphate backbone (Figure 2B), while the arginines (R168 and R172) interact with the cytosine base in the -4 position (Figure 2B), and the side chain of Y176 inserts between two bases (Figure 2B). Together, these interactions position the DNA substrate and ensure that the catalytic tyrosine hydroxyl is at the required distance from the scissile phosphate in the active site for DNA cleavage to take place. Concurrently, the interactions of R168 and R172 with the cytosine base in the –4 position contribute to the preference or specificity of DNA cleavage sites. Analysis of the site-directed alanine substitution mutations of the EcTOP1 residues required for the interactions with the cytosine in the –4 position showed that these mutants have significantly decreased DNA cleavage activity, and that the preference of the cytosine in the –4 position can be altered to an adenine base instead (38).

A lysine conserved in topoisomerase III enzymes (K8 in EcTOP3) has been proposed to replace the role of the metallic cation required by type IIA topoisomerase in the active site (23). A lysine is conserved in bacterial topoisomerase I in a nearby, but non-equivalent position (K13 in EcTOP1, K28 in MtbTOP1) as shown in Supplementary Figure S1. Site-directed mutagenesis of EcTOP1 K13 showed that this lysine is required for DNA cleavage (39), but its positively charged side chain is not in the vicinity of the scissile phosphate in any EcoTOP1 crystal structures available to date. We also find that K28 is not near the DNA phosphates in the active site of MtbTOP1-704t bound to DNA in the absence or presence of metal ions (Figure 3). We cannot dismiss the possibility that K28 of MtbTOP1 and its equivalent lysine in bacterial topoisomerase I can move into the active site to interact with the transition state in a reaction intermediate that has not been captured by crystallography. It is also possible that the role of K13 in EcTOP1 and K28 in MtbTOP1 in catalysis is different from K8 in EcTOP3, as their positions are not equivalent (Supplementary Figure S1). The lysine conserved in this specific position of bacterial topoisomerase I may be required for the assembly of the active site. It may also have a polarizing effect on the nearby acidic residues, as previously proposed (39).

Certain mutants of EcTOP1 and MsmTOP1 with specific substitutions at the conserved arginine and aspartates in the active site have been demonstrated to be dominant lethal (53). These mutant enzymes can still cleave DNA while DNA rejoining in the presence of Mg^2+^ became less efficient, resulting in accumulation of covalent intermediates and bacterial cell death (47,51,53). MtbTOP1 is essential for the viability of the TB pathogen, and can be exploited as a novel target for discovery of new therapies (1,2,54,55) in the treatment of MDR-TB. Genetic manipulation to deplete MtbTOP1 and inhibition of MtbTOP1 activity by small molecules have been shown to inhibit growth (2,54). Significant bactericidal activity may require only a small number of the trapped covalent topoisomerase-DNA intermediates (56) through the action of topoisomerase poison inhibitors (11,12). Small molecules that can selectively perturb the MtbTOP1 active site interactions in the presence of Mg^2+^ observed in the structure of enzyme/DNA/Mg complex reported here may be candidates for topoisomerase poison inhibitors against MtbTOP1.

## DATA AVAILABILITY

Atomic coordinates and structure factors for the reported crystal structures have been deposited with the Protein Data bank under accession numbers 5UJ1, 5UJY, 6CQ2 and 6CQI.

## Supplementary Material

Supplementary Data
