# Supplementary material for: Investigating mycobacterial topoisomerase I mechanism from the analysis of metal and DNA substrate interactions at the active site
Source: Nucleic Acids Res. 2018 Jun 14;46(14):7296–308. doi: 10.1093/nar/gky492 (PMC6101483; doi:10.1093/nar/gky492)
Supplement: Supplementary Data [file gky492_supplemental_files.zip › SI_NAR.pdf]

Supplementary Information

Methods

Crystallization screening. The screening for crystallization conditions was performed with the help of a Mosquito nanoliter liquid handler (TTP LabTech) using the sitting-drop vapor diffusion technique in 96-well CrystalQuick plates (Greiner). For each condition, 0.4 μl of protein and 0.4 μl of crystallization formulation were mixed; the mixture was equilibrated against 140 μl of the crystallization solution in each reservoir well. The crystallization screens used were MCSG-1–4 (Microlytic) Index and PEG/Ion HT^{TM} (Hampton Research) at 16 °C. Predominantly thin needle-like or hair-like crystals appeared under multiple conditions.

Crystallization Conditions

A) MtbTOP1-704t /MTS2-11

PEG/Ion HT^{TM} screen, D8 condition

0.2 M ammonium phosphate dibasic pH 8.0; 20% (w/v) polyethylene glycol 3,350.

B) MtbTOP1-704t/MTS2-13/Mg

MCSG-4 screen, E12 condition

0.1 M sodium acetate, 0.1 M MES:NaOH pH 6.5; 30% (w/v) PEG 2000 MME.

C) MtbTOP1-704t /MTS2-13/Mg (2nd crystal form)

MCSG-2 screen, H2 condition

0.2 M potassium chloride, 0.05 M HEPES:NaOH pH 7.5,

35% (v/v) pentaerythritol propoxylate (5/4PO/OH).

D) MtbTOP1-704t (high resolution)

MCSG-2 screen, A1 condition

0.2 M potassium sodium tartrate, 20% (w/v) PEG 3350.

E) MtbTOP1-704t (2nd crystal form)

MCSG-1 screen, G1 condition

0.1 M HEPES:NaOH pH 7.5, 10% (w/v) PEG 8000, 8% (v/v) ethylene glycol

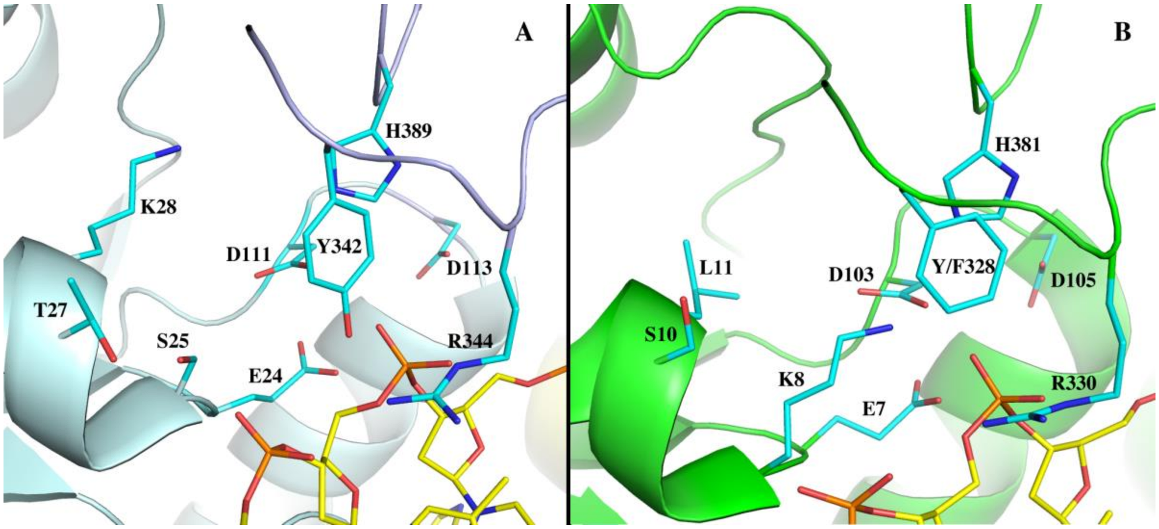

Figure S1. Comparison of the active sites of *M. tuberculosis* topo I and *E coli* topo III in pre-cleavage state. Close up views of the active sites of (A) MtbTOP1-704t/ssDNA complex (PDB code: 6CQ1); (B) EcTOP3-Y328F/ssDNA complex (PDB code: 1I7D). Key residues and DNA ssDNA are drawn in stick format.

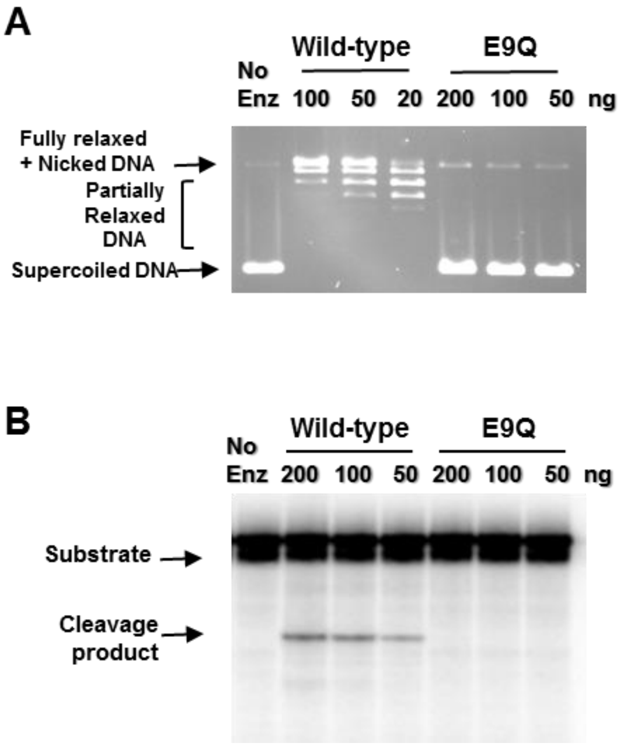

Figure S2. E9Q mutant of *E. coli* topoisomerase I has null relaxation and DNA cleavage activity (A) Relaxation assay: Agarose gel electrophoresis of products from incubation of supercoiled pBAD/Thio plasmid DNA with indicated amounts of wild-type and E9Q mutant enzyme at 37°C for 30min. (B) ssDNA cleavage assay: 59-base oligonucleotide 5'-GCCCTGAAAGATTATGGAATGCGATTAGGGTAAAGGAAGAGAGCATAATCTTTCAGGGC-3' labeled with ^{32}P at the 5'-end was incubated with indicated amount of enzyme at 37 °C for 30 min. The substrate and product were separated by electrophoresis in a 15% sequencing gel.

SI : Morph conformations

Movie #1

A trajectory that morphs MtbTOP1-704t from its conformation in apo form (PDB code: 5UJ1) to its conformation in a holo form in the structure of MtbTOP1-704t /MTS2-11 (PDB code: 6CQI). The ssDNA in the structure of MtbTOP1-704t /MTS2-11 is not shown.

Movie #2

The apo form MtbTOP1-704t (PDB code: 5UJ1) is initially shown in a structural alignment with the holo form MtbTOP1-704t /MTS2-11 (PDB code: 6CQI). The third domain (D3) from each structure is used for the alignment (see text). The movie shows how the apo form MtbTOP1-704t morphs to the holo form MtbTOP1-704t /MTS2-11 upon DNA binding.

Both morph movies were made with UCSF Chimera program (1).

Reference

1. Pettersen E.F., Goddard T.D., Huang C.C., Couch G.S., Greenblatt D.M., Meng E.C. and Ferrin T.E. (2004) UCSF Chimera—A Visualization System for Exploratory Research and Analysis. *J Comput Chem*. **25**, 1605-12)
